# Characterization of the Frmd7 Knock-Out Mice Generated by the EUCOMM/COMP Repository as a Model for Idiopathic Infantile Nystagmus (IIN)

**DOI:** 10.3390/genes11101157

**Published:** 2020-09-30

**Authors:** Ahmed Salman, Samuel B. Hutton, Tutte Newall, Jennifer A. Scott, Helen L. Griffiths, Helena Lee, Diego Gomez-Nicola, Andrew J. Lotery, Jay E. Self

**Affiliations:** 1 Clinical and Experimental Neurosciences, University of Southampton, Southampton SO16 6YD, UK; T.Newall@soton.ac.uk (T.N.); j.a.scott@soton.ac.uk (J.A.S.); h.l.griffiths@soton.ac.uk (H.L.G.); helena.lee@soton.ac.uk (H.L.); A.J.Lotery@soton.ac.uk (A.J.L.); jes3@soton.ac.uk (J.E.S.); 2 School of Psychology, University of Sussex, Brighton BN1 9QH, UK; sam@sr-research.com; 3 School of Biological Sciences, University of Southampton, Southampton SO171BJ, UK; d.gomez-nicola@soton.ac.uk

**Keywords:** nystagmus, Idiopathic Infantile Nystagmus, FRMD7, retina, optokinetic nystagmus

## Abstract

In this study, we seek to exclude other pathophysiological mechanisms by which *Frmd7* knock-down may cause Idiopathic Infantile Nystagmus (IIN) using the *Frmd7^.tm1a^* and *Frmd7^.tm1b^* murine models. We used a combination of genetic, histological and visual function techniques to characterize the role of *Frmd7* gene in IIN using a novel murine model for the disease. We demonstrate that the *Frmd7^.tm1b^* allele represents a more robust model of *Frmd7* knock-out at the mRNA level. The expression of *Frmd7* was investigated using both antibody staining and X-gal staining confirming previous reports that *Frmd7* expression in the retina is restricted to starburst amacrine cells and demonstrating that X-gal staining recapitulates the expression pattern in this model. Thus, it offers a useful tool for further expression studies. We also show that gross retinal morphology and electrophysiology are unchanged in these *Frmd7* mutant models when compared with wild-type mice. High-speed eye-tracking recordings of *Frmd7* mutant mice confirm a specific horizontal optokinetic reflex defect. In summary, our study confirms the likely role for *Frmd7* in the optokinetic reflex in mice mediated by starburst amacrine cells. We show that the *Frmd7^.tm1b^* model provides a more robust knock-out than the *Frmd7^.tm1a^* model at the mRNA level, although the functional consequence is unchanged. Finally, we establish a robust eye-tracking technique in mice that can be used in a variety of future studies using this model and others. Although our data highlight a deficit in the optiokinetic reflex as a result of the starburst amacrine cells in the retina, this does not rule out the involvement of other cells, in the brain or the retina where *Frmd7* is expressed, in the pathophysiology of IIN.

## 1. Introduction

Nystagmus is a condition of the eye characterized by involuntary and uncontrolled eye movements, initiated by abnormal slow phases away from the visual target [1,2]. The prevalence of nystagmus in the general population is estimated to be 24 per 10,000 [3]. Nystagmus can be congenital, typically occurring in the first 6 months of life, or acquired [4,5]. Over 40 different types of nystagmus have been described and differ according to onset, pathology, and nature of the eye movement [2,4,6]. Infantile Nystagmus Syndrome (INS) is a form of nystagmus that classically presents between 4 and 6 months of age, stays horizontal in vertical gaze, has accelerating slow phases and may be associated with abnormal head postures due to null zones and dampening on convergence [7]. Idiopathic Infantile Nystagmus (IIN) is occurring in a patient with no other identifiable ocular or visual pathway disorder. Nystagmus can have a significant impact on vision. Many patients with the condition score lower in visual function questionnaire studies than those with Age-related Macular Degeneration (AMD) [7,8]. 

IIN has no detectable association with a visual sensory defect, although it is genetically heterogeneous and has been described as an autosomal dominant [9], autosomal recessive, and X-linked dominant trait [10], most cases are not familial, many with no association with a specific allele. However, The FERM (F for 4.1 protein, E for ezrin, R for radixin and M for moesin) domain-containing 7 (*FRMD7*) gene is the only gene which is known to be associated with IIN and accounts for approximately 70%, of known X-linked IIN cases [11]. Eye movement recordings reveal that IIN has a predominantly horizontal jerk waveform, although pendular nystagmus has been seen in both adults and infants with *FRMD7* mutations [12,13], with a defect in the horizontal, but not the vertical, optokinetic reflex (OKR, and sometimes called OKN) was suggested [14]. OKN is an involuntary reflex initiated by a visual scene drifting on the retina, triggering the eyes to perform a slow movement in the direction of the visual scene followed by fast corrective saccades in the opposite direction. OKN works with the vestibulo-ocular reflex (VOR) in which eye movement stimulated by head or body motion while animals move their entire head in pursuit of moving object [15]. The status of OKN in IIN is still open to debate. 

The *FRMD7* gene is a member of the Band 4.1 superfamily. They were identified and named from a Coomassie-stained protein gel [16]. They are a group of proteins characterized by a conserved domain known as FERM domain (4.1/ezrin/radixin/moesin). This is conserved among the band 4.1 superfamily of proteins, and interacts with both plasma membrane and cytoskeleton. The number of identified FERM-containing proteins is rapidly increasing, with 50 different proteins characterized into three major groups: (i) talins and kindlins, (ii) ERMs (ezrin/radixin/moesin), GEFs (guanine-nucleotide-exchange factors), kinases and phosphatases, and finally (iii) myosins and KRITS (krev interaction trapped proteins) [17]. The FERM domain is a cysteine-rich hydrophobic module of approximately 300 amino acids. A partial crystal structure of FERM-containing motif for ezrin [18], moesin [19,20] and radixin [21,22] has been shown and these proteins have diverse functional roles in cell signaling events including organization of the cytoskeleton and cell proliferation [23,24,25,26]. Several expression sites of *FRMD7* have previously been reported in human embryonic tissues including the brain and the developing neural retina. *FRMD7* expression has also been reported in human adult kidney, liver and pancreas, and at low levels, heart and brain [11,27]. *Frmd7* expression has also been reported in the starburst amacrine cells of the murine retina [14], brain [28], hippocampus, cerebellum, cortex and Olfactory bulb [29]. The function of the *FRMD7* protein is not yet fully understood; however, it is believed to be involved in signal transduction between plasma membrane and cytoskeleton similar to other FERM domain-containing proteins, such as FERM RhoGEF and pleckstrin domain-containing protein 1 and 2 (*FARP1* and *FARP2* respectively). The presence of the ezrin/radixin/moesin proteins, which are known to regulate cell adhesion and morphogenesis, in the *FRMD7* protein supports current hypothesis on its association with the plasma membrane of neurons, which might act as a guidance mechanism for their growth [30,31,32]. Recently, loss of *Frmd7* has been reported to abolish neuronal circuit asymmetry resulting in direction selectivity defects in the murine retina [14] and result in loss of horizontal OKR. However, the mechanism of this effect is still unknown. 

The *Frmd7* conditional-knock-out (*Frmd7^.tm1a^*) model, where the *Frmd7* coding sequence has been distributed resulting in a non-functional protein, is the first reporter model of *Frmd7,* the presence of the *LacZ* gene in the targeting cassette, which is driven by the *Frmd7* promoter, provides a tool to characterize sites of *Frmd7* expression. Although *Frmd7^.tm1a^* mice have been reported to harbor a deficit in the OKN in the horizontal direction [14], the potential uses of the model have not been fully explored. Here, we use *Frmd7^.tm1a^* mice to explore expression patterns of *Frmd7* and characterize its function in IIN. We confirm previously reported expression sites of Frmd7 in the starburst amacrine cells of the mouse retina using a combination of detection methods and reveal X-gal staining as an alternative, and more robust, method to detect Frmd7 expression than antibody staining. We also confirm the horizontal OKN deficit in these mice using a robust and reliable eye-tracking methodology. in addition, we examined the electrical activity of the retina by electroretinography (ERG) and the integrity of the retinal layers by ocular coherence tomography (OCT). We show that deleting the critical exon in the *Frmd7^.tm1a^* mice, which generates the *Frmd7^.tm1b^* mice, despite creating a more robust *Frmd7* knock-down at the mRNA level, has no effect on the severity of the optokinetic defect. 

## 2. Materials and Methods 

### 2.1. Animals

We confirm adherence to the Association for Research in Vision and Ophthalmology (ARVO) Statement for the Use of Animals in Ophthalmic and Vison Research in our experimental design. All animal procedures were performed in accordance with standard ethical guidance guidelines (European Communities Guidelines on the Care and Use of Laboratory Animals, 86/609/EEC) and were approved by the Home Office (UK) and the University of Southampton ethical committee under animal license number 30/3189. Wild-type (C57BL/6) mice were obtained from Charles River. *Frmd7 ^tm1a^* mice refer to homozygous females or hemizygous males *Frmd7 ^tm1a(KOMP)Wtsi^* mice (https://www.sanger.ac.uk/science/collaboration/mouse-resource-portal). A splice acceptor *IRES-LacZ* targeting cassette was inserted between exon 3 and exon 4 (the critical exon), designed to produce a truncated N-terminal Frmd7 protein (68-amino acids) and β-galactosidase (the product of the *LacZ* gene). Unless otherwise indicated, animals were between postnatal day 9 (P9) and P120 and maintained in C57BL/6 background. *Frmd7* expression was reported to be at peak during early postnatal development between P8 and P9 in mice [14]. P120 represents an adult retinal developmental stage before any age-related retinal degeneration might occur. 

### 2.2. Confocal Microscopy

Immunohistochemistry signals in wholemount retinas were analyzed using SP8 Confocal system (LEICA, Wetzlar, Germany). Signals were assessed from 1024 × 1024-pixel z-stack images at 0.5 μm z steps taken with 63× glycerol or 40× oil immersion lens. Images were processed using Fiji software, for quantification of fluorescence intensity profile images were converted to 8-bit type then intensity threshold was measured in black and white.

### 2.3. DAB (3,3′-diaminobenzidine) Staining

Frozen sections were dried for 30 min at 37 °C, post-fixed for 10 min in absolute alcohol then washed in 0.1% Tween^TM^ 20 in PBS (PBST). Endogenous hydrogen peroxidase activity was quenched in 0.3% Hydrogen peroxidase (Sigma-Aldrich) and 10% methanol in 0.1% PBST for 30 min. Sections were then washed in 0.1% PBST and blocked in 5% normal goat serum (NGS; Sigma-Aldrich) and 5% BSA in 0.2% PBST for 1 h at room temperature. Primary antibodies were incubated overnight at 4 °C in 5% NGS, 5% BSA in 0.2% PBST, then incubated with secondary antibodies for 1 h at room temperature in 5% NGS, 5% BSA in PBST. Hydrogen peroxidase activity was detected by avidin/biotin detection method using VECTASTAIN^®^ Elite ABC (Avidin-Biotin) detection system (VECTOR Laboratories, Peterborough, UK). Sections were subjected to 3,3′-diaminobenzidine (DAB) substrate prepared with 0.3% hydrogen peroxidase for appropriate time, before dehydrating through ethanol series to 100% ethanol. Sections were then mounted in Dibutylphthalate Polystyrene Xylene (DPX) mounting solution (MilliporeSigma, St.Louis, MO, USA).

### 2.4. Electroretinography 

ERGs were recorded using Generation II Image-Guided ERG attachment to the Micron IV Retinal imaging system (Phoenix Research Labs, California, CA, USA). Dark adaptation performed since the rods are the predominant type of photoreceptors in the rodent retina. Mice were dark adapted for 1 h at 25 °C under the UK Home Office regulation procedure guidance. For pupil dilation, a drop of 1% Tropicamide eye drop solution (Bausch and Lamb Laboratories, UK) was administered on the surface of the eye, followed directly by a drop of 2.5% Phenylephrine hydrochloride eye drop solution (Bausch and Lamb Laboratories, Bridgewater, NJ, USA). Viscotear liquid gel (Alcon Laboratories, Geneva, Switzerland) was applied at regular basis (every 30 min) to avoid corneal drying. Three electrodes were attached, a ground electrode attached to the base of the tail, a reference electrode attached subcutaneously between the ears and a gold-tipped ring corneal electrode (which attached to the Micron IV camera) is making contact with the cornea. ERGs were recorded by stimulation for 1 min with white light flash (6.8 cd-s/m^2^) illuminated by the LED light component of the Generation II image-Guided ERG system with a duration of 1 milli-second with 2 sweeps of 2-min intervals in between. Recordings were stored then analyzed with Microsoft Excel and GraphPad Prism7 software packages. 

### 2.5. Head Fixation for Eye-Tracking Studies

Head fixation was achieved by surgical attachment of a metal plate to mice skull and mice were then attached to a metal plate under general anesthesia. The metal plate was surgically fixed by exposing by making a small excision of skin between the ears, groves made at the surface of the skull with a sterile razor blade and a small metal bolt (M1.0 × 2 SLOT CHEESE MACHINE SCREW DIN 84 A2 Stainless Steel) (Precision Technology Supplies) was fixed to the skull with a drill, then an L-shaped metal plate was glued with Histoacryl Tissue Adhesive (Blue) (Williams Medical). Exposed skull was covered with Simplex Rapid Powder and liquid dental cement (Kemdent; ISO 20795) according to the manufacturer’s instructions. 

### 2.6. Immunofluorescence Staining

Whole mount retinas were dissected and fixed for 30 min in 4% paraformaldehyde (PFA), for cryosectioning, eyes were dissected and cryoprotected in sucrose overnight before embedding in OCT. Frozen sections were dried for 30 min at 37 °C, post-fixed in absolute alcohol (Thermo Fisher Scientific, Waltham, MA, USA) before being blocking in 5% normal donkey serum (NDS) and 5% BSA in 0.2% PBST for 1 h at room temperature. For wholemount staining, retinas were blocked immediately after fixation. Primary antibodies were incubated over night at 4 °C in 5% NDS and 5% BSA in 0.2 PBST, then incubated with secondary antibodies for 1 h at room temperature. Section were incubated in 20 μm 4′,6-diamidino-2-phenylindole (DAPI) for 5 min before mounting in Mowiol^®^ 4-88 Mounting Reagent (Calbiochem) prepared with 0.1% Citifluor AF4 Antifadent Mountant Solution (Agar scientific) according to standard procedures.

### 2.7. Eye Tracking

Eye tracking was performed with EyeLink 1000 eye-tracking system (SR Research Ltd., Ottawa, ON, Canada). Although the system may have been “designed with human eye tracking in mind” it will track any eye capable of providing a pupil and corneal reflection and has been used in tracking eyes of non-human primates and dogs. To adapt the system for eye tracking in rodents we used a 50 mm lens with a 2 mm c-ring spacer to increase the size of the image of the eye and reduce the focal distance. Some internal parameters were changed to accommodate differences in the relative size of the pupil/cr in the mouse eye compared to human eye. The EyeLink 1000 works by illuminating the eye with infra-red (IR) light. A video camera films the eye at 1000Hz (1000 frame per second), and the location of the pupil and corneal reflection in each image are determined. Visual stimulation, controlled by Experiment Builder software (SR Research Ltd., Ottawa, ON, Canada), was presented on two computer monitors placed in a ‘V’ position in front of the mouse for monocular stimulation. Recordings were performed on head-fixed mice to minimize head movement. For motion stimulation (OKN) black and white gratings at different sizes, thin (2.5 degrees), medium (5.3 degrees) and wide (21 degrees), moved at ~5.3°/s. One degree corresponds to 31 μm retinal distance in mice [33]. The OKN stimulus was present (in motion) the entire time during recording. The raw data recorded by the eye tracker is in arbitrary x,y, units, and as it is uncalibrated. The system did not need calibrating since we were simply looking for the presence/absence of OKN rather than measuring the amplitude and other quantifiable parameters. To analyze the data, each recording (all of which are 10 s long) was rescaled by subtracting the mean x value for the 10 s of data from each of the data points within the recording. Control wild-type and *Frmd7^.tm1a^* and *Frmd7^.tm1b^* mice (*n* = 4 per group) were subjected to black and white gratings in the following order: horizontal left, horizontal right, vertical up, and vertical down with 1 min per stimulus direction and 2 min rest between stimuli. OKN response was mainly observed when subjected to medium size gratings. The OKN was quantified using RStudio software, for each stimulus direction the number, duration, start time and finish time and number of beats recorded were quantified and compared to that of the control mice to determine whether or not an OKN was present.

### 2.8. Light Microscopy

Immunohistochemistry signals of anti-choline acetyltransferase (ChAT) DAB stained and X-gal stained sections were analyzed using the OLUMPUS BX51 microscope equipped with ‘.Slide’ OLYMPUS Soft Imaging Solutions GmbH camera (OLYMPUS, Shinjuku, Japan). Slides were scanned and imaged with 40× or 63× oil immersion lens, images were then analyzed with Fiji and Adobe Photoshop software packages. 

### 2.9. Ocular Coherence Tomography (OCT)

Tomograms from individual mice eyes were acquired using Envisu R2200 VHR SDOIS Mouse Imaging system (Bioptigen, Durham, NC, USA). Following ERG recordings, anesthetized mice were placed in a holding rotator, so a single spot of light is shined into the center of the eye. Images were centered around the optic nerve and 1.4 mm scans were taken through 50 degrees. Retinal images were then segmented using InVivoVue 2.4 Diver automated analysis software using 5 × 5 grid segmentation across the retina, each point of the grid segmented to the following layers: Retinal Nerve Fiber Layer (RNFL), outer RNFL, Inner Plexiform Layer (IPL), Inner Nuclear Layer (INL), Outer Plexiform Layer (OPL), Outer Nuclear Layer (ONL), Inner Segment Ellipsoid (IS), Outer ISE (OS), end tips (EPTRS) and Retinal Pigment Epithelial layer (RPE). Thickness of layers in *Frmd7^.tm1a^* and *Frmd7^.tm1b^* compared to those of wild-type controls using Microsoft Excel and Prism7 software packages. 

### 2.10. Quantitative Polymerase Chain Reaction (PCR)

Total ribonucleic acid (RNA) was extracted from wild-type, *Frmd7.^tm1a^* and *Frmd7.^tm1b^* retinae using ambion Trizol reagent (Thermo Fisher Scientific) following a standard protocol. To form cDNA, RNA was reverse transcribed using random hexamers and the iScript^TM^ Reverse Transcription for RT-qPCR system (BioRad) according to the manufacturer’s instructions. *Frmd7* splice variants were determined from wild-type, *Frmd7.^tm1a^* and *Frmd7.^tm1b^* cDNA by PCR amplification using the iTaq^TM^ Universal *N′,N′*-dimethyl-*N*-[4-[(E)-(3-methyl-1,3-benzothiazol-2-ylidene)methyl]-1-phenylquinolin-1-ium-2-yl]-*N*-propylpropane-1,3-diamine (CYBR) Green Supermix (BioRad; 170-5124), and the following primers combination used to amplify the different transcripts: P1 (ex2F/ex3R), P2 (ex3F/ex4R), P3 (ex3F/ex5R), P4 (ex2F/ex5R), P5 (ex3F/En2R), P7 (LacZF1/LacZR2) (Supplementary Table S1), according to manufacturer instructions. 

### 2.11. Statistical Analysis

Statistical analyses were performed using GraphPad Prism Software (GraphPad, San Diego, CA, USA). The statistical significances of differences were determined by the unpaired student’s *t*-test when data were unpaired and skewed. if the data were normally distributed and paired, then a parametric test was used (ANOVA). Data are expressed as means ± standard error of the mean (SEM) with statistical significance denoted as * for *p* ≤ 0.05 and ** for *p* ≤ 0.01. 

### 2.12. X-gal Staining

Frozen retinal sections were fixed in cold X-gal fix (1% formaldehyde, 0.2% glutaraldehyde (Sigma–Aldrich)) for 10 min at 4 °C then washed 3 times for 10 min in X-gal buffer (5 mM EGTA (pH 8.0), 2 mM MgCl, 0.04% NP-40, 0.01% deoxycholate acid (Sigma–Aldrich) in PBS, pH 7.9), before staining with X-gal stain (5 mM potassium hexacyanoferrate (III) (K3Fe), 5 mM potassium ferricyanide (K4Fe), 0.5% X-gal substrate (Fisher Scientific) in X-gal buffer) at 37 °C overnight following an in-house optimized protocol. Briefly, X-gal buffer was prepared by adding 0.045 g (K3Fe), and 0.06 g (K4Fe) in 25 mL X-gal buffer. 250 μL X-gal substrate was added to the mix shortly before staining. Sections were incubated with X-gal stain at 37 °C in the dark overnight. Sections were washed 3 times in X-gal buffer and post-fixed in 4% PFA for 1 h at 4 °C. Sections were dehydrated through a graded ethanol series and cleared in a CLERENE Solvent (LEICA) twice for 5 min. Sections were immediately mounted in DPX mounting medium.

## 3. Results

### 3.1. Quantification of the Transcript Variants from Frmd7^.tm1a^ and Frmd7^.tm1b^ Alleles Reveals That the Frmd7^.tm1b^ Allele Provides a More Robust and Efficient Knock-Down of Frmd7 Expression 

**Figure 1:**
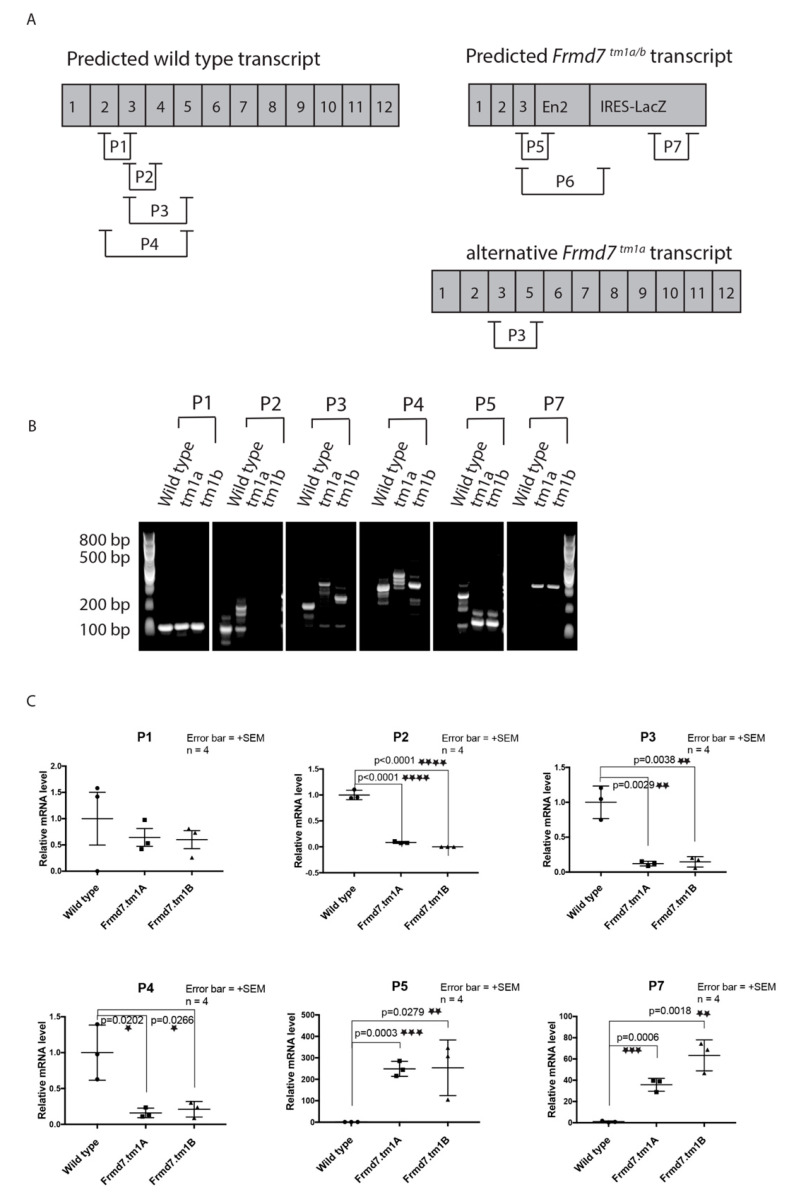
Schematic diagram showing the different *Frmd7* mRNA transcript variants in wild-type, *Frmd7^.tm1a^* and *Frmd7^.tm1b^* mice. (**A**) Schematic representation of the predicted mRNAs in wild-type, *Frmd7^.tm1a^* and *Frmd7^.tm1b^* mice (*n* = 4 each), with primer combinations (P1–P7) amplifying across the mRNA transcript. Numbers represents corresponding exons (1–12), En2 and IRES-*LacZ* are part of the *LacZ*-tagged targeting cassette used to generate the *Frmd7^.tm1a^* allele. (**B**) Agarose gel electrophoresis of RT-PCR products in wild-type, *Frmd7^.tm1a^* and *Frmd7^.tm1b^* male mice with predicted band sizes with the different combination of primers used in RT-PCR. (**C**) Relative mRNA expression levels for wild-type, *Frmd7^.tm1a^*, *Frmd7^.tm1b^* and wild-type male mice with different combination of primers (P1–P7). Stars refer to statistical significance. Error bars = +SEM, significance of *p* values calculated using a parametric ANOVA test. See qPCR full gel in Figures S5 and S6.

The *Frmd7^.tm1b^* mice were generated by using the actions of the Cre recombinase as the critical exon (exon 4) was originally flanked by *LoxP* sites in the *Frmd7^.tm1a^* allele designed by the EUCOMM/KOMP resource (see Supplementary Figure S1). RT-PCR analysis of wild-type, *Frmd7^.tm1a^* and *Frmd7^.tm1b^* transcripts revealed a difference in the relative mRNA expression between the *Frmd7^.tm1a^*, which contained significant wild-type transcript, and the *Frmd7^.tm1b^*, that lacked any wild-type transcript (Figure 1). PCR amplification across each exon of the Frmd7 transcript is explained in Figure 1A. The PCR products shown in Figure 1B shows the following: (i) The P1 primer combination amplifies across exon 2 and exon 3, which is present in the wild-type transcript. (ii) P2 primer combination was used to examine the presence of exon 4 excised transcript. (iii) Primer combination P3 and P4 were used to determine whether the mRNA transcript is full as in the wild-type mice or truncated as expected in the *Frmd7^.tm1a^* and *Frmd7^.tm1b^* mice. (iv) Primer combinations P5–P7 amplify across the *LacZ*-driven targeting cassette and only present in the mutant mice. Those combination of primers were used to distinguish between transcripts present in both *Frmd7* mutant and wild-type mice. The relative mRNA levels for the different primer combinations are shown in Figure 1C.

### 3.2. Frmd7 Expression Is Specific to the Starburst Amacrine Cells of the Murine Retina

**Figure 2:**
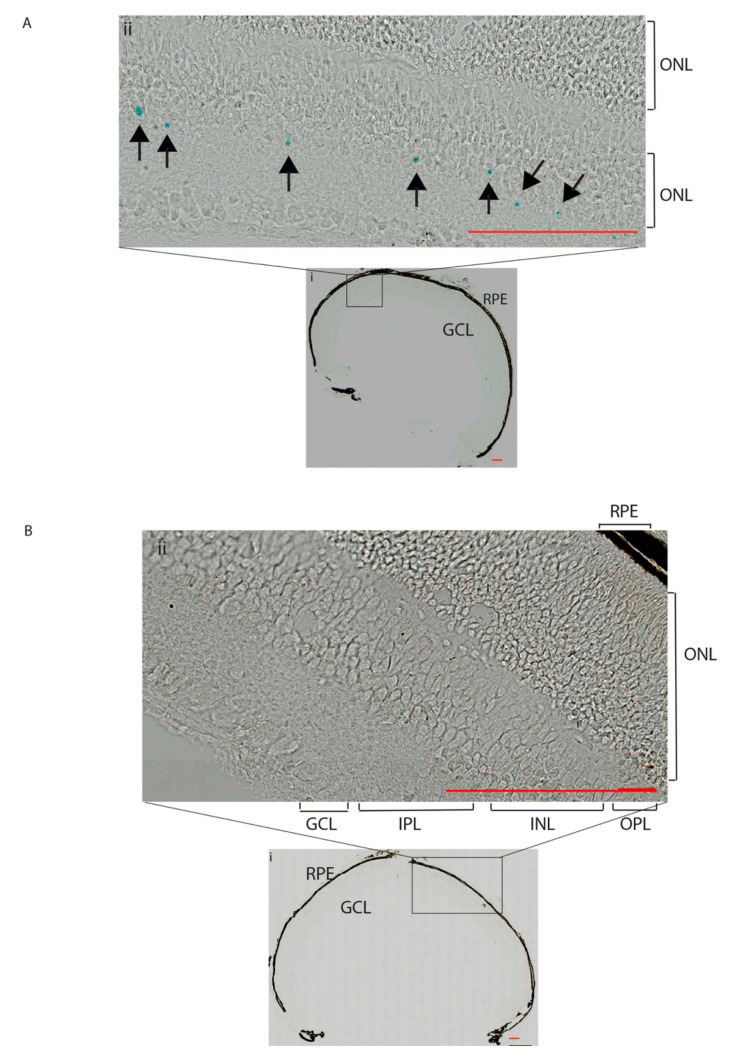
*Frmd7*-driven *LacZ* expression in *Frmd7^.tm1a^* mice at P9. Frozen retinal sections of P9 *Frmd7^.tm1a^* hemizygous males (**A**) and wild-type littermates (**B**) (*n* = 3) with different lateral sections of the sagittal plane (Magnification = 63×, scale bar = 100 μm for all images). The eyes were dissected, and the cornea and lens were removed leaving the back of the eye with the retina (i), with zoomed in area (ii). The different layers of the retina are labelled starting from the inner to the outer retina as follows: the GCL (Ganglion Cell layer), the IPL (Inner Plexiform Layer), the INL (Inner Nuclear Layer), the Outer Plexiform layer (OPL), the ONL (Outer Nuclear Layer) and the Retinal Pigment Epithelium (RPE). LacZ expression can be seen in the GCL and INL (arrows) as previously reported [14].

**Figure 3:**
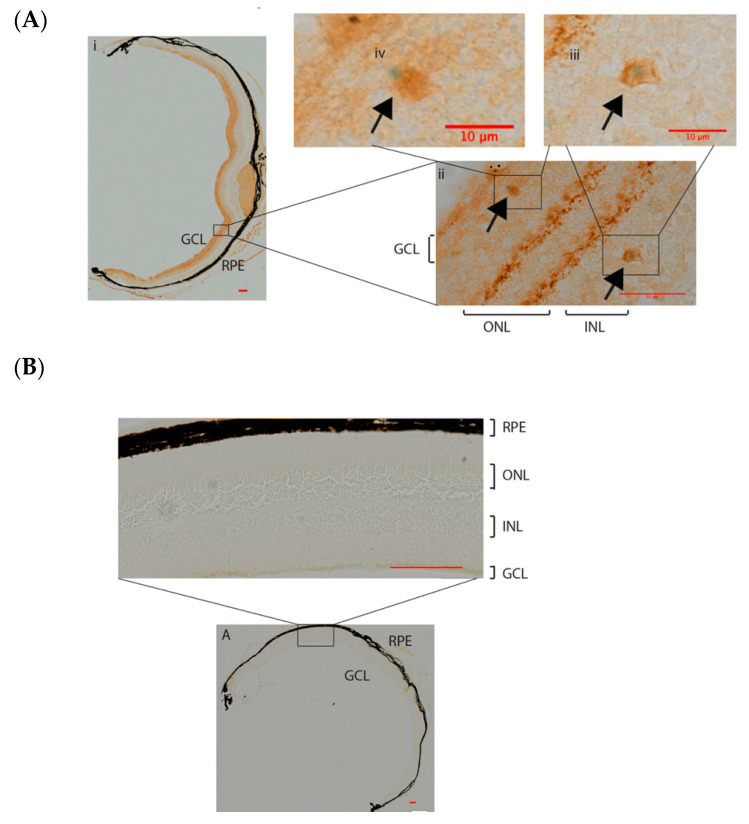
Colocalization of *Frmd7* promoter-driven *LacZ* expression and ChAT proteins in *Frmd7^.tm1b^* retina. Frozen sections from adult *Frmd7^.tm1b^* retina (*n* = 3) subjected to X-gal staining first then to DAB staining with an anti-Choline acetyltransferase ChAT antibody (**A**). Sections from C57/BL6 retinas subjected to X-gal staining then to DAB staining with the anti-ChAT antibody were used as negative controls (**B**). Arrows indicate colocalization between *LacZ* staining (blue), which is driven by the *Frmd7* promoter, and ChAT proteins in the Ganglion Cell Layer (GCL) and the Inner Nuclear Layer (INL). Scale bar = 100 μm unless otherwise indicated.

We obtained further evidence that *Frmd7* expression is restricted to the starburst amacrine cells of the murine retina [14]. *Frmd7* expression was reported to be at peak during early postnatal developmental stages (P), between P8 and P9 mice [14]. β-galactosidase, the product of the *LacZ* gene, is driven by the *Frmd7* promoter in the *Frmd7^.tm1a^* mice. X-gal staining of frozen retinal sections of P9 *Frmd7^.tm1a^* mice show specific expression of *Frmd7* in the starburst amacrine cells in the neonatal (P9) murine retina (Figure 2A), where X-gal staining can be seen in the ganglion cell layer (GCL) and INL. No staining was observed in the wild-type retina (Figure 2B). *Frmd7* expression was maintained in the GCL and INL of the adult retina (Supplementary Figure S2). No difference in the distribution of the *Frmd7*-positive cells between the central and peripheral retina was observed. To ensure that the X-gal staining, which represents *Frmd7* expression, is truly associated with the starburst amacrine cells, frozen sections of *Frmd7^.tm1a^* retina stained with X-gal and then co-stained with choline acetyltransferase (ChAT) antibody, which is a starburst amacrine cell marker (Figure 3). *Frmd7* expression appears to be associated with ChAT-positive cells, specifically in the cell body of the GCL and the INL. No staining was observed in the wild-type retina (Figure 3B). Immunohistochemistry analysis of retinal sections from wild-type mice using antibodies against ChAT and Frmd7 proteins show similar colocalization between ChAT and Frmd7 proteins in the GCL and INL [14]. To rule out a defect in the synaptic connectivity of the ChAT expressing cells in the *Frmd7* mutant retinas, we also examined the density of the starburst amacrine cells and evaluated the synaptic intensity of several synaptic markers in adult (P120) retinas. P120 represents an adult retinal developmental stage before any age-related retinal degeneration might occur. Synaptic markers included choline acetyltransferase (ChAT), which is an amacrine cells marker; synaptophysin, a presynaptic marker; post-synaptic density 95 (PSD95), a post-synaptic marker; vesicular choline acetyltransferase (VAChT) to track ChAT packaging into vesicles; glutamic acid decarboxylase (GAD 65/67), a marker for gamma aminobutyric acid (GABA), and vesicular GABA transferase (VGAT) for vesicular packaging of GABA, (See Supplementary Figure S3), with no obvious abnormality in the levels of expression of all markers. 

### 3.3. Presence of Low Levels of Frmd7 Wild-Type Transcript in the Frmd7^.tm1a^ Did Not Result in Detectable Frmd7 Protein 

**Figure 4:**
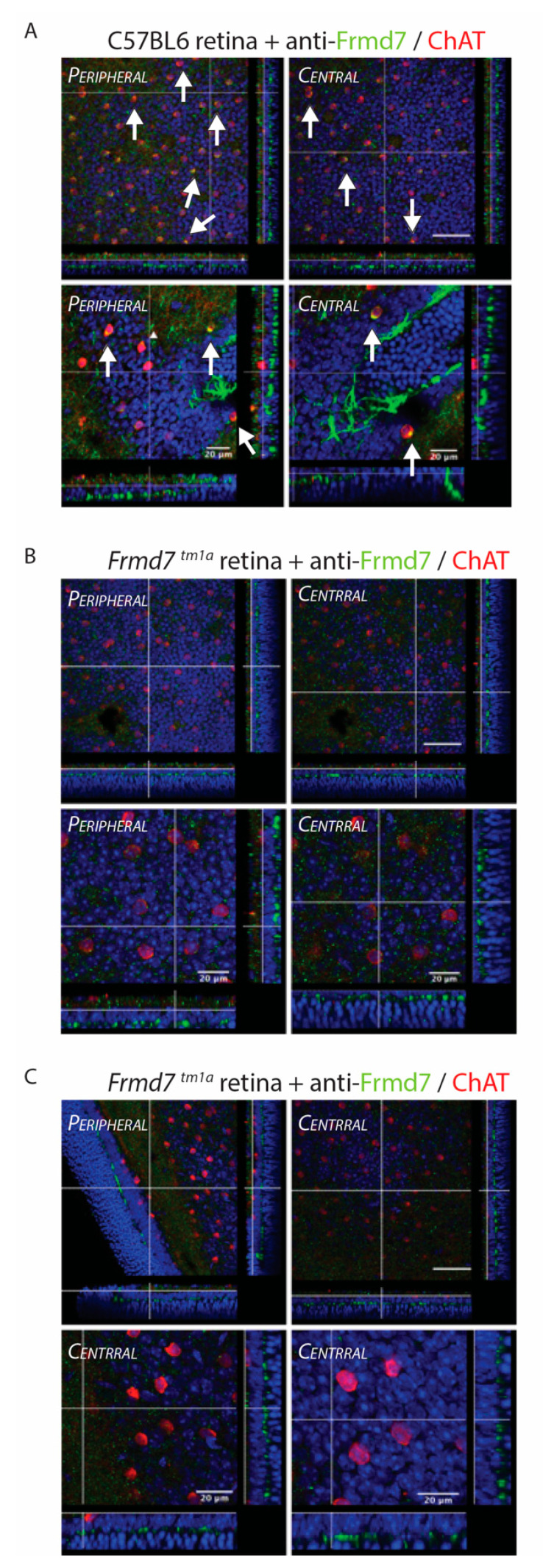
Immunofluorescence staining of adult *Frmd7^.tm1a^*, *Frmd7^.tm1b^* and wild-type wholemount retinas. Confocal images of wholemount retinas of wild-type littermates (*n* = 3) (**A**), *Frmd7^.tm1a^* (*n* = 3) (**B**) and *Frmd7^.tm1b^* (*n* = 3) (**C**), were subjected to immunofluorescence staining with the anti-Frmd7 antibody raised against the murine Frmd7 protein (FITC) and an anti-Choline acetyltransferase (ChAT) antibody (red) and shown in top view. Side views are shown on the bottom and right-hand sides. White arrows indicate Frmd7 and ChAT proteins colocalization. (4′,6-diamidino-2-phenylindole) (DAPI) (blue) was used as a counterstain. Scale bar = 100 μm unless otherwise indicated.

It is unknown whether the presence of low levels of wild-type transcript in the *Frmd7^.tm1a^* mice would result in detectable levels of protein. Immunohistochemistry analysis of wholemount and frozen retinal sections of adult (P120) wild-type, *Frmd7^.tm1a^* and *Frmd7^.tm1b^* mice stained with anti-ChAT and anti-*Frmd7* antibodies reveals colocalization between ChAT (red) and Frmd7 (green) proteins in the wild-type retina (Figure 4A). No obvious colocalization between ChAT and Frmd7 proteins can be seen in both *Frmd7^.tm1a^* and *Frmd7^.tm1b^* retinas (Figure 4B,C respectively and Supplementary Figure S4), indicating the presence of (low levels of) wild-type *Frmd7* transcript in the *Frmd7^.tm1a^* mice does not translate to a detectable level of protein. The colocalization between ChAT and Frmd7 proteins seen in the wild-type retina is not present in both *Frmd7^.tm1a^* and *Frmd7^.tm1b^* retinas. Staining has been observed in the OPL specifically to neurites, this pattern of staining has not been previously reported and it is likely to be as a result of non-specific binding of the anti-murine Frmd7 antibody. However, no colocalization with ChAT can be seen in this layer as ChAT-positive cells only exist in the INL and the GCL.

### 3.4. Frmd7 Knock-Out Mice Show No Detectable Defect in the Electrical Activity of the Retina

**Figure 5:**
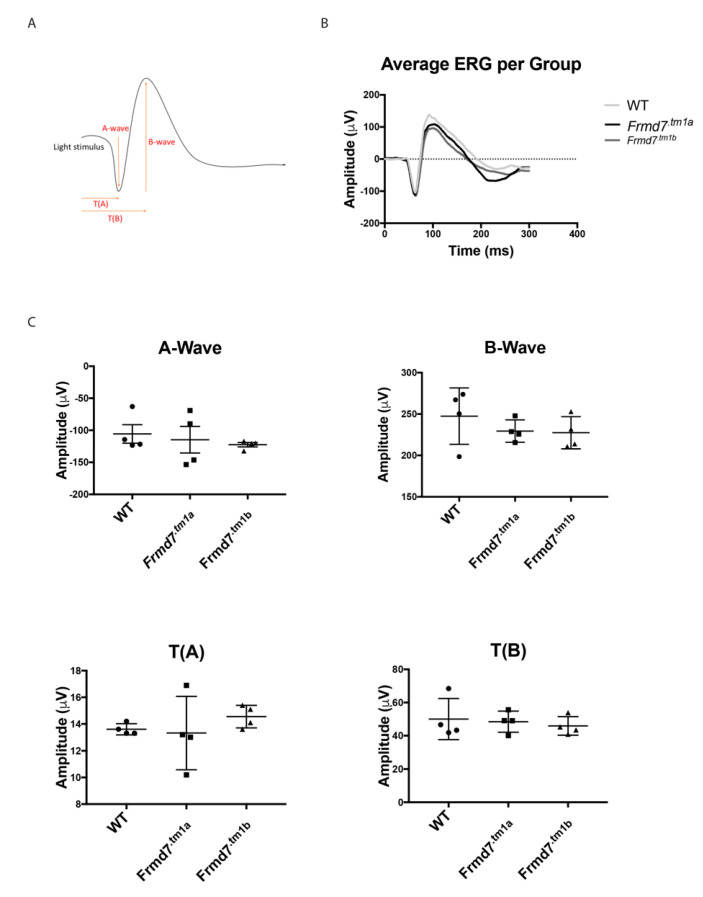
Electroretinogram (ERG) analysis of *Frmd7^.tm1a^*, *Frmd7^.tm1b^* and wild-type mice. Statistical analysis of ERGs of wild-type, *Frmd7^.tm1a^* and *Frmd7^.tm1b^* mice showing a typical murine ERG wave with the A-wave, B-wave indicating the duration and direction of each wave (**A**). A representative figure of the average ERG waves of adult (10 weeks) *Frmd7^.tm1a^* and *Frmd7^.tm1b^* and wild-type littermate mice (*n* = 4) (**B**). Dot plots representing no significant statistical change in A-wave per mouse group (*p* = 0.290, *p* = 0.308 and *p* = 0.185 respectively), B-wave (*p* = 0.234, *p* = 0.691 and *p* = 0.443 respectively), T(A) (*p* = 0.161, *p* = 0.709 and *p* = 0.563 respectively) and T(B) (*p* = 0.055, *p* = 0.632 and *p* = 0.413 respectively) (**C**). Error bars = +SEM. Significance of *p* values calculated using a parametric ANOVA test.

Electroretinography (ERG) analysis on a murine model of *Frmd7* knock-out has not been previously reported. To assess the electrical activity of retinas of adult (P120) *Frmd7^.tm1a^*, *Frmd7^.tm1b^* mice in comparison to wild-type mice, we recorded ERG’s on dark adapted mice and revealed no differences in the average ERG wave, represented by both the A-wave (the initial negative deflection) and the B-wave (the subsequent rise towards the positive peak) (Figure 5A,B). Average amplitudes of the A-wave the B-wave appears to be similar that of the mutant mice (*Frmd7^.tm1a^* and *Frmd7^.tm1b^*), as well as the average times of the A-wave and B-wave (T(A) and T(B) respectively) between the different groups (Figure 5C). P120 represents an adult retinal developmental stage before any age-related retinal degeneration might occur. 

### 3.5. Optical Coherence Tomography (OCT) Scans of Frmd7 Knock-Out Mice Demonstrate Normal Retinal Morphology

**Figure 6:**
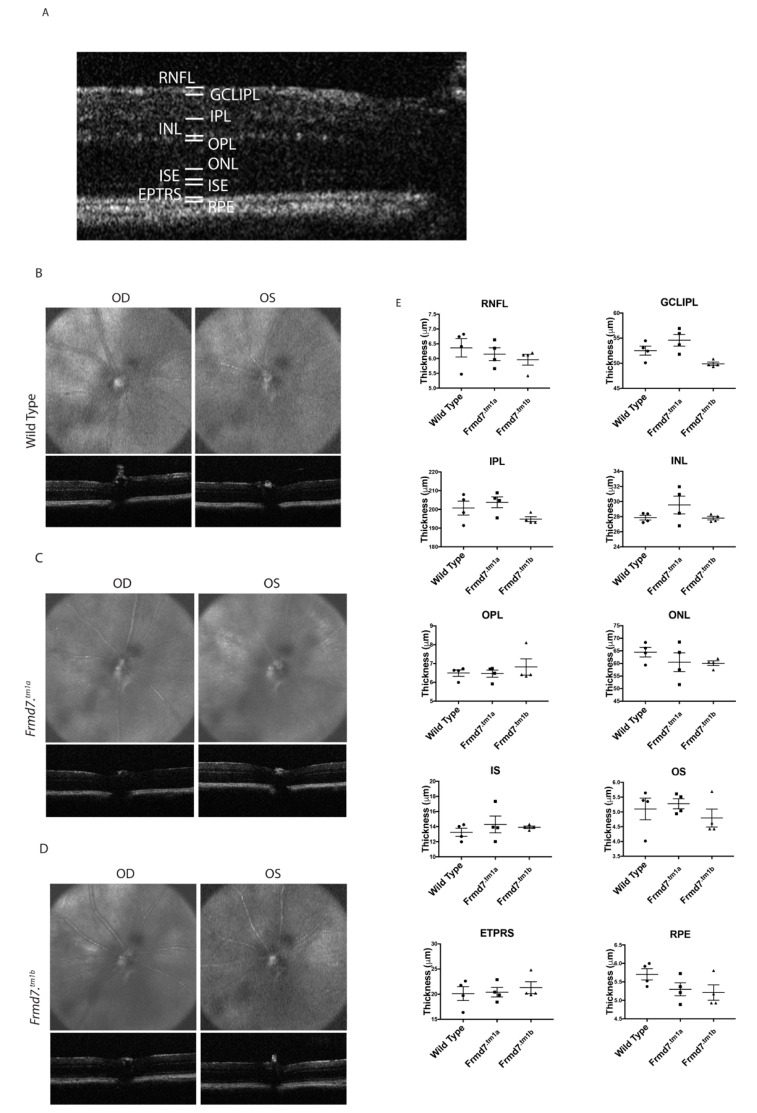
Ocular Coherence Tomography (OCT) analysis of *Frmd7^.tm1a^*, *Frmd7^.tm1b^* and wild-type mice. Schematic representation of the different layers of the retina showing the boundaries used to measure each layer (**A**). OCT scans of oculus dextrus (OD) and oculus sinister (OS) in full views (top) and side views (bottom) of wild-type littermate (**B**) *Frmd7^.tm1a^* (**C**) and *Frmd7^.tm1b^* (**D**) adult (10 weeks) male mice (*n* = 4). (**E**) Statistical analysis of the different layers of the retina for each group showing no significant difference in the thickness of each layer Retinal Nerve Fiber Layer (RNFL, *p* = 0.3565), Ganglion Cell Layer (GCLIPL, *p* = 0.159), Inner Plexiform Layer (IPL, *p* = 0.1842), Inner Nuclear Layer (INL, *p* = 0.2072), Outer Plexiform Layer (OPL, *p* = 0.9572), Outer Nuclear Layer (ONL, *p* = 0.4677), Inner Segment Ellipsoid (IS, *p* = 0.8919), Outer ISE (OS, *p* = 0.6968), end tips (EPTRS, *p* = 0.8012) and Retinal Pigment Epithelial layer (RPE, *p* = 0.1247). Error bars = +SEM. Significance of p values calculated using a parametric ANOVA test.

Characterizing changes to the thickness of the retina is a routine examination to determine changes to the retina because of conditions with ocular defects. To determine the thickness of the retinal layers of *Frmd7^.tm1a^* and *Frmd7^.tm1b^* mice in comparison to the wild-type, we have taken ocular coherent tomogram (OCT) scans from each group immediately after electroretinography (ERG) recordings took place. The boundaries between the different layers of the retina (Figure 6A) were used analysis of gross morphology change in the retina. The OCT scans of adult (P120) wild-type, *Frmd7^.tm1a^*, *Frmd7^.tm1b^* retinas (Figure 6B–D respectively) showed no obvious significant changes in the thickness of the different layers of the retina of all groups. Statistical analysis of the thickness of the retinal layers including the RNFL, ganglion cell layer (GCLIPL), IPL, INL, OPL, ONL, IS, outer segment (OS), end tips (EPTRS) and RPE revealed no significant change in the thickness of the layers measured (Figure 6E). Similarly IIN individuals with *Frmd7* mutations show no changes in the thickness of retinal layers [2]. This result is consistent with the assumption that retinal thickness is unaffected by *Frmd7* in the murine retina as is observed in humans with *FRMD7* mutations for extra-foveal retinal regions, which suggests that the *Frmd7^.tm1a^* and *Frmd7^.tm1b^* mice are not abnormal, similar to the phenotype in individuals with IIN.

### 3.6. Frmd7 Knock-Out Mice Show a Deficit in the Horizontal Optokinetic Reflex 

**Figure 7:**
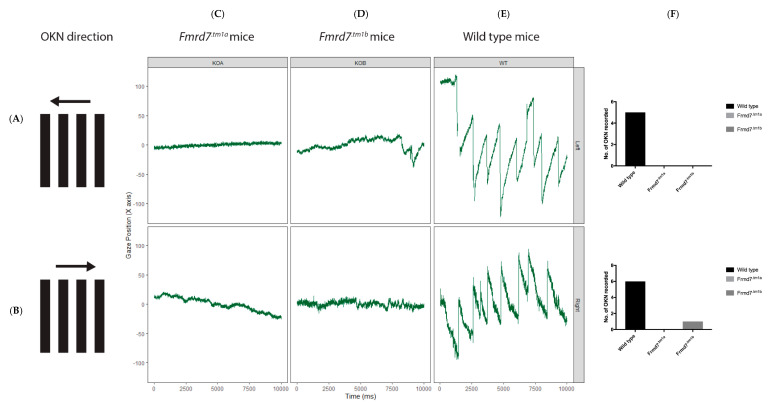
Horizontal OKN analysis of *Frmd7^.tm1a^*, *Frmd7^.tm1b^* and wild-type mice. Eye-tracking recordings of wild-type (**E**), *Frmd7^.tm1a^* (**C**) and *Frmd7^.tm1b^* (**D**) in response to motion a continuous stimulus (OKN) to the *nasotemporal* (**A**) and *temporonasal* (**B**) directions on the retina. Black bars represent motion stimulus direction, bars were moving at 5.3 degree per second. (F) Bar charts on the right represent quantification of the number of horizontal optokinetic reflexes (OKN) counted for each group, Eye-tracking recordings on the horizontal left direction were as follows; for wild-type mice (*n* = 5), 8 recordings (6 had shown OKN), *Frmd7^.tm1a^* mice (*n* = 5), 9 recordings (none had shown OKN), *Frmd7^.tm1b^* mice (*n* = 5), 6 recordings (none had shown OKN). For eye-tracking recordings on the horizontal right direction; wild-type mice, 8 recordings (5 had shown OKN), *Frmd7^.tm1a^* mice, 8 recordings (none had shown OKN), *Frmd7^.tm1b^* mice, 7 recordings (only 1 had shown OKN). Error bars = +SEM. Significance of p values calculated using a parametric ANOVA test.

We compared the OKN, in head-fixed adult (p120) *Frmd7^.tm1a^*, *Frmd7^.tm1b^* and wild-type mice (Figure 7). Eye-tracking recordings of wild-type mice elicited a strong OKN in the horizontal (temporonasal and nasotemporal) direction. Similar to human subjects with IIN that lack horizontal OKN [34], the *Frmd7^.tm1a^ Frmd7^.tm1b^* mice lacked an OKN in the horizontal direction too, response was mainly observed using medium size gratings (5.3 degrees). We observed no spontaneous oscillatory eye movements (nystagmus) in both the *Frmd7^.tm1a^ Frmd7^.tm1b^* mice. Since the raw data recorded by the eye tracker is in arbitrary x,y, units (uncalibrated), it is not possible to convert units of measurements into degrees of visual angle and the figure is an illustrative. To make the recordings comparable (for illustrative purposes), each recording (all of which are 10 s long) was rescaled by subtracting the mean × value for the 10 s of data from each of the data points within the recording. Eye movement recordings in response to moving gratings in the vertical direction were inconsistent and unsuitable for robust analysis and are not reported. These results show that a common symptom, the lack of horizontal optokinetic reflex, is shared between humans and mice lacking functional *FRMD7* protein, and that the mechanism of eye movement control in mice and humans is similarly sensitive to FRMD7 loss.

## 4. Discussion

The *Frmd7^.tm1a^* targeted allele is the first reporter and conditional allele for the *Frmd7* gene. Expression sites of the human and murine *Frmd7* gene have been described in various reports in the literature and a combination of in vivo and in vitro functional studies have been previously reported [11,14,29,35,36,37]. The methods used to characterize *Frmd7* expression sites were limited to analyses such as immunohistochemistry using antibodies against the Frmd7 protein and wholemount in situ hybridization (WISH) using mRNA probes. Previously reported expression analyses for *Frmd7* are inconsistent partly due to the lack of a reliable antibody against the murine Frmd7 protein and the low sensitivity of mRNA probes used for WISH [27,29]. In this study, we have explored the potential advantages of this *Frmd7* knock-out mouse model to further characterize *Frmd7* expression patterns and function in order to study human *FRMD7* mutant nystagmus. The generation of the *Frmd7^.tm1b^* mice by Cre recombinase activity to delete the critical exon (exon 4) in the *Frmd7^.tm1a^* allele showed that differences exist between different splice variants of the *Frmd7* knock-out allele (Figure 1). The *Frmd7^tm1b^* transcript lacked the wild-type transcript present in the *Frmd7^tm1a^* allele, suggesting that the *Frmd7^tm1b^* model may be more robust for future studies. However, our preliminary studies did not identify a clear difference between the models regarding phenotype. 

We have explored the reliability of using X-gal staining as a method to study *Frmd7* gene expression patterns using retinas from *Frmd7^tm1a^* mice. We confirm previously reported sites of *Frmd7* expression in the starburst amacrine cells of the murine retina [14] (Figure 2) and show that X-gal staining will be a useful tool in the further study of this model.

The mechanism by which *Frmd7* modulates signaling of the starburst amacrine cells is still unknown. However, it has been suggested that Frmd7 might be involved in modulating signaling between the plasma membrane and the actin cytoskeleton dynamics through interactions with Rho GDP-dissociation inhibitor alpha, the main regulator of Rho GDPases [36,38]. Further study is clearly required to understand the role of the Frmd7 protein in the developing retina.

An additional unanswered question is whether the dysfunction of the starburst amacrine cells that seems to lead to a deficit in the OKN seen in the *Frmd7^.tm1a^* and *Frmd7^.tm1b^* mice is also present in human subjects with *FRMD7* mutations [34,39]. Several pieces of evidence support the case that this may indeed by true. Current evidence suggests that the neuronal pathways controlling the OKN are conserved across mammals. The ON DSGCs that are horizontally selective, and are activated by the NOT/DNT signals, have been recorded by NOT/DNT in primate brains, although it is important to note that in primates there are also projections into the NOT/DNT pathway from cortical motion areas MT/MST (middle temporal area and medial superior temporal area respectively) [40], which over complicates the rodent/human connection. Also, choline acetyltransferase (ChAT), which is an amacrine cell marker, shows similar expression patterns in non-human primate and mice retina, where the cell bodies of the starburst amacrine cells lie in the INL and the GCL and two mosaics of starburst cells processes extend into the strata of the IPL [14,41]. In addition, *Frmd7* mRNA expression was detected in the same layers in the non-human retina [14]. This indicates that in primates *FRMD7* is expressed in cells that are morphologically and genetically similar to the starburst amacrine cells in mice. The motor system controlling horizontal eye movements seems to be functional in human subjects with IIN and the *Frmd7^.tm1a^* mice since both show normal spontaneous horizontal eye movements [14]. These findings contribute to the hypothesis that loss of horizontal OKN is, at least partly, due to the loss of *Frmd7* function in the starburst amacrine cells. It is also notable that *FRMD7* mRNA has been detected in brain regions associated with the VOR [11,34]. However, human studies do not seem to suggest that abnormalities of the VOR underlie IIN in human subjects. In contrast to IIN human subjects with *FRMD7* mutations, both *Frmd7^.tm1a^* and *Frmd7^.tm1b^* mice did not show spontaneous horizontal oscillations (nystagmus), this can be due to the possibility that the lack of horizontal OKN is not caused by the same mechanism as nystagmus. Those possibility needs examining to shed more light on the mechanisms of IIN.

Most mutations in X-linked IIN were found in the FERM and the FA adjacent domains. The role of *FRMD7* in IIN is not yet understood; however, the involvement of the FERM domain and the FA adjacent domain in regulating plasma membrane and actin cytoskeleton organization is believed to be the mechanism behind *FRMD7* regulation of developing neural system, and loss of *FRMD7* can cause a defect in neurite development in the region of the brain controlling eye movement [35,36].

## 5. Conclusions

In this study, we have characterized and compared two *frmd7* mutant alleles (*Frmd7^.tm1a^* and *Frmd7^.tm1b^*) which represent the only current murine models of IIN. We have shown no difference in ERG or OCT parameters between the two strains and wild-type mice. The lack of oscillatory potential in the mutant mice, as well as the wild-type mice, does not seem to be related to the starburst amacrine cells. It would be interesting to examine the oscillatory potential of the B-wave to evaluate the electrical activity of the starburst amacrine cells in the *Frmd7* mutant mice. OCT scans of the mutant mice reveal no difference in the thickness of retinal layers compared to the wild-type mice. This result is consistent with the assumption that retinal thickness is unaffected by *Frmd7* in the murine retina as observed in humans with *FRMD7* mutations for extra-foveal retinal regions. This cannot be assumed for the foveal region as mice are afoveate species. In addition to the ERG and OCT findings, we confirmed a specific horizontal OKR deficit in both using novel eye-tracking methods. We have shown differences in the mRNA expression pattern for *Frmd7* between the two mutant strains with the *Frmd7^.tm1a^* allele expressing significant amounts of wild-type transcript. We also demonstrate no apparent consequence of this difference at the protein level or through phenotyping using OCT, ERG, and eye-tracking techniques. However, we suggest that for future studies which may be more susceptible to low levels of coexisting wild-type transcript, the *Frmd7^.tm1b^* allele is likely to offer a more robust model.

## Supplementary Materials

The following are available online at https://www.mdpi.com/2073-4425/11/10/1157/s1, Figure S1: a schematic diagram showing the *Frmd7^.tm1b^* targeted allele by PCR verification. Figure S2: representative images of *Frmd7*-driven *LacZ* expression in adult *Frmd7^.tm1a^* mice. Figure S3: a representation of the density of synaptic markers in the mouse retina. Figure S4: w images showing wholemount retina immunofluorescence staining of *Frmd7*-expressing starburst amacrine cells. Figure S5: qPCR full gel for Figure 1 (transcript)—P1, P3, P4 and P7. Figure S6: qPCR full gel for Figure 1 (transcript)—P2 and P5. Table S1: PCR primers table and finally full gel images of qPCR.
