# Supplementary material for: Characterization of the Frmd7 Knock-Out Mice Generated by the EUCOMM/COMP Repository as a Model for Idiopathic Infantile Nystagmus (IIN)
Source: Genes (Basel). 2020 Sep 30;11(10):1157. doi: 10.3390/genes11101157 (PMC7601595; doi:10.3390/genes11101157)
Supplement: Supplementary file 1 [file genes-11-01157-s001.pdf]

Supplementary Materials: Characterization of the Frmd7 Knock-Out Mice Generated by the EUCOMM/COMP Repository as a Model for Idiopathic Infantile Nystagmus (IIN)

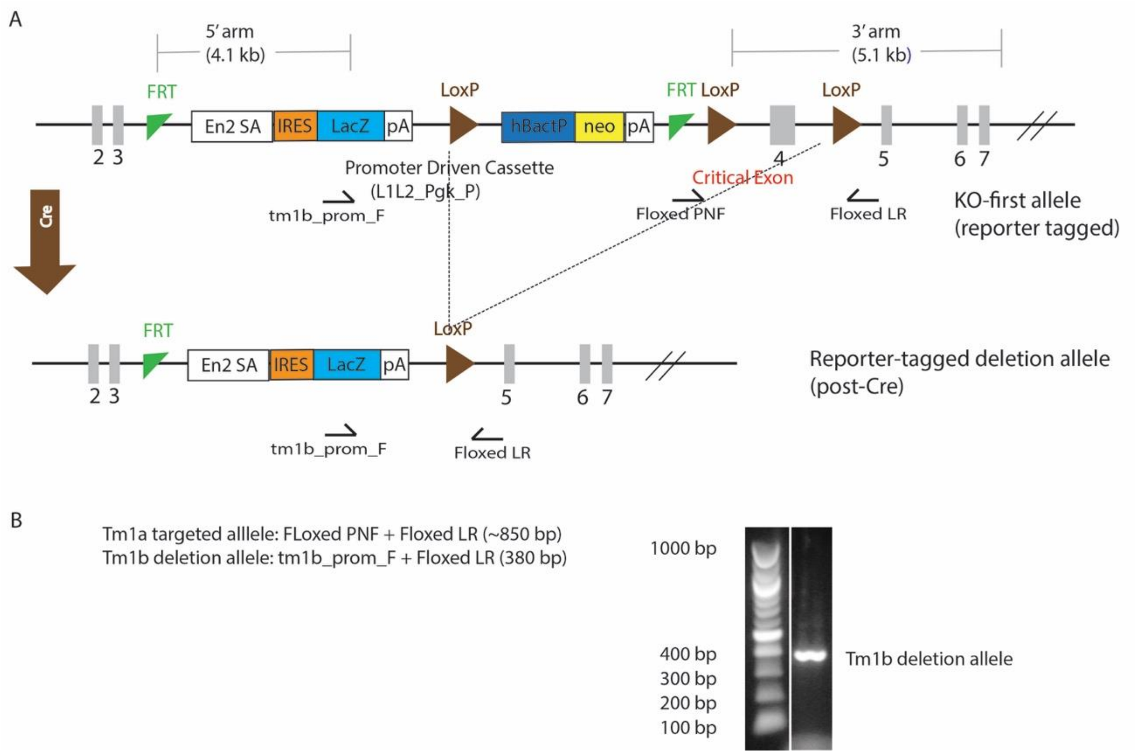

Figure S1. Schematic diagram showing the *Frmd7.tm1b* targeted allele by PCR verification. (A) The *Frmd7.tm1a* EUCOMM/KOMP targeted allele contains a *LacZ* reporter sequence (blue boxes) and neomycin resistance selection cassette (yellow boxes) upstream of the *Frmd7* critical exon (grey boxes) on the 5′ targeting arm (grey). (B) PCR genotyping of a progeny from homozygous *Frmd7tm1b* female offspring. The primers (black arrows) amplified ~380 bp product, representing the *Frmd7.tm1b* deletion allele.

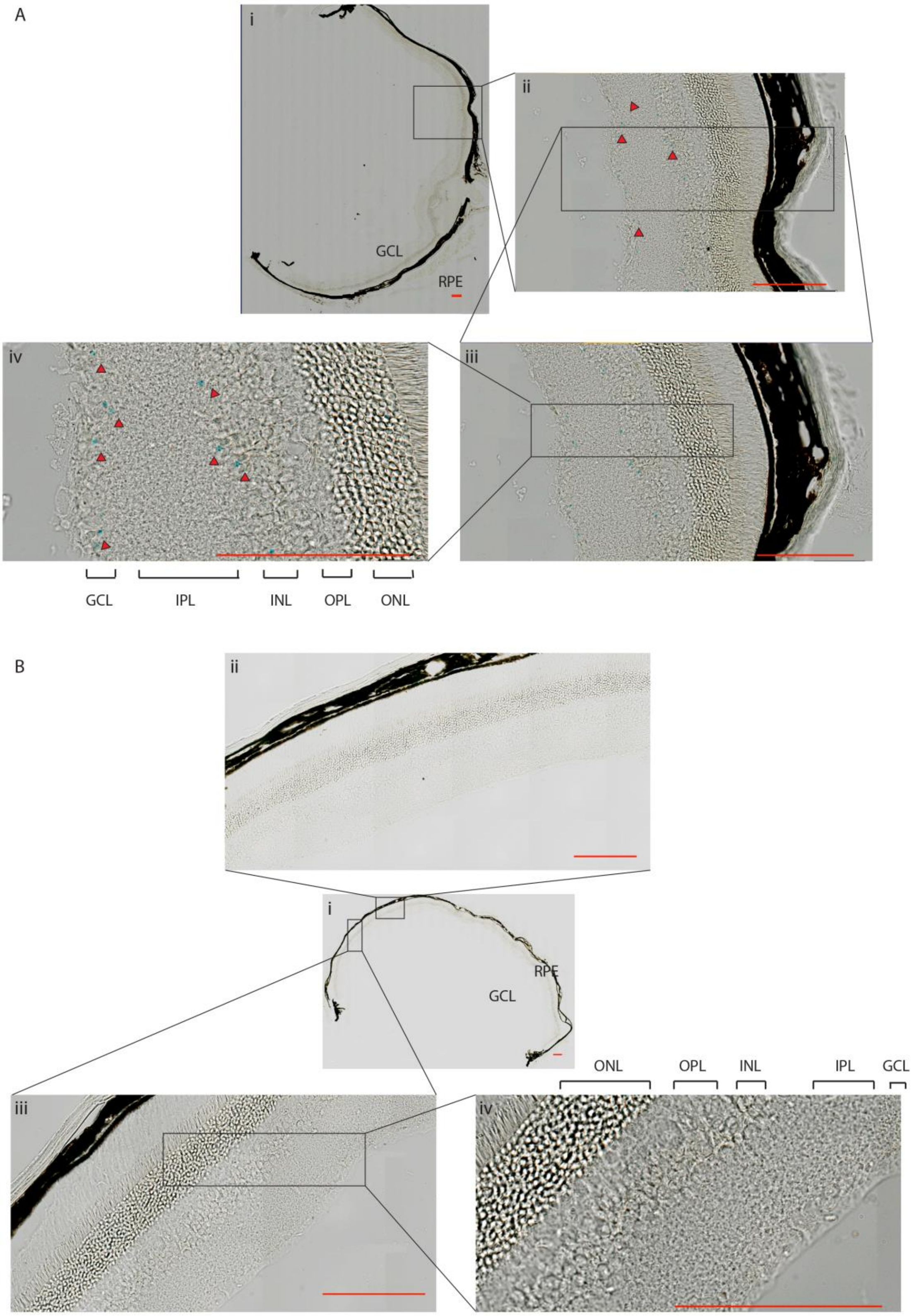

Figure S2. *Frmd7*-driven *LacZ* expression in adult *Frmd7*^{tm1a} mice. Frozen retinal sections of adult (10 weeks) *Frmd7*^{tm1a} hemizygous males (A) and wild type littermates (B) (n=3) with different lateral sections of the sagittal plane (Magnification = 63x, scale bar = 100 µm for all images). The cornea and the lens were dissected leaving the back of the eye with the retina (i), with zoomed in areas (ii, iii, and iv respectively). The different layers of the retina are labelled starting from the inner to the outer retina as follows: the GCL (Ganglion Cell layer), the IPL (Inner Plexiform Layer), the INL (Inner Nuclear Layer),

the Outer Plexiform layer (OPL), the ONL (Outer Nuclear Layer) and the Retinal Pigment Epithelium (RPE). LacZ expression can be seen in the GCL and INL (arrows) as previously reported [25].

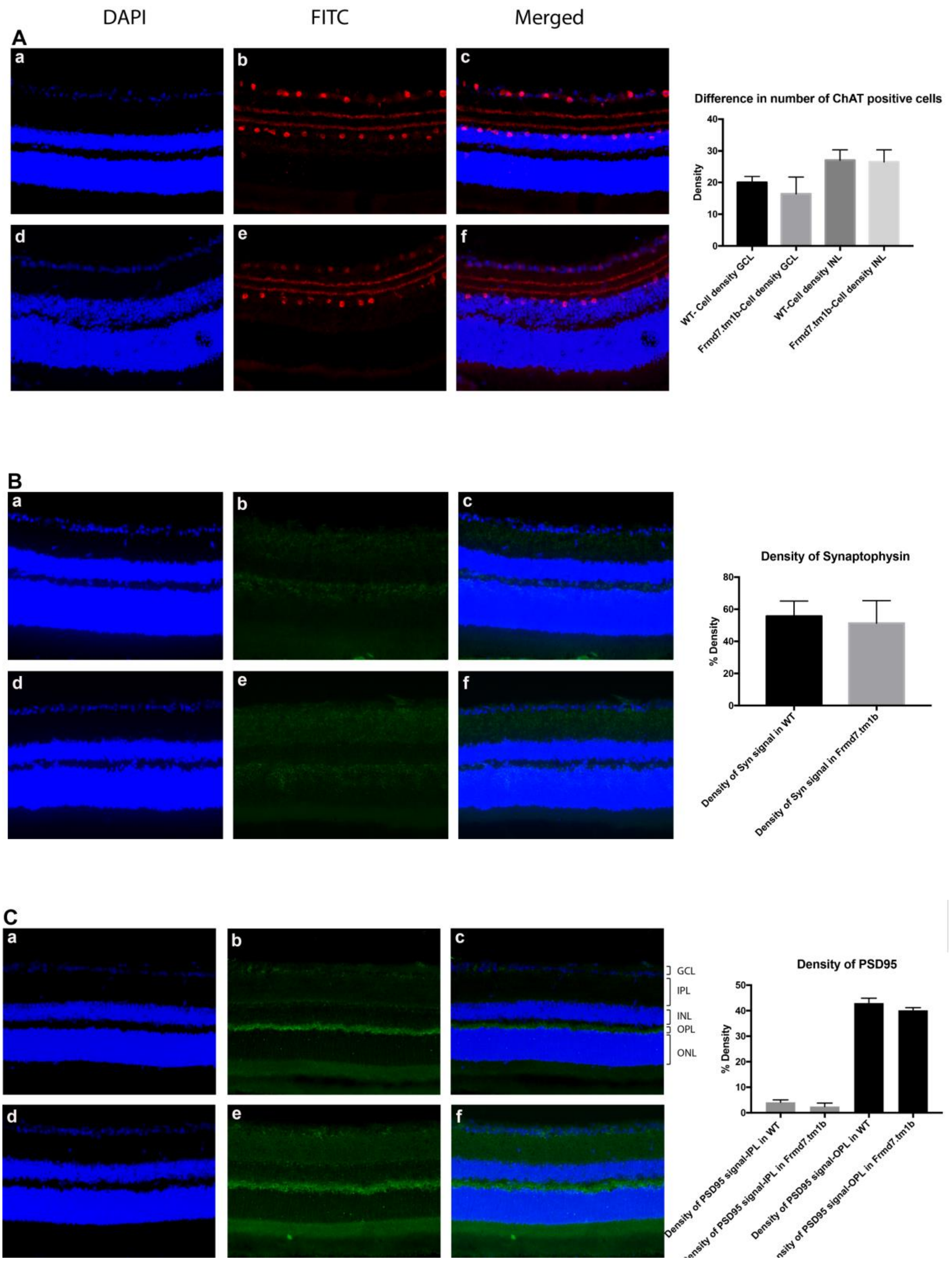

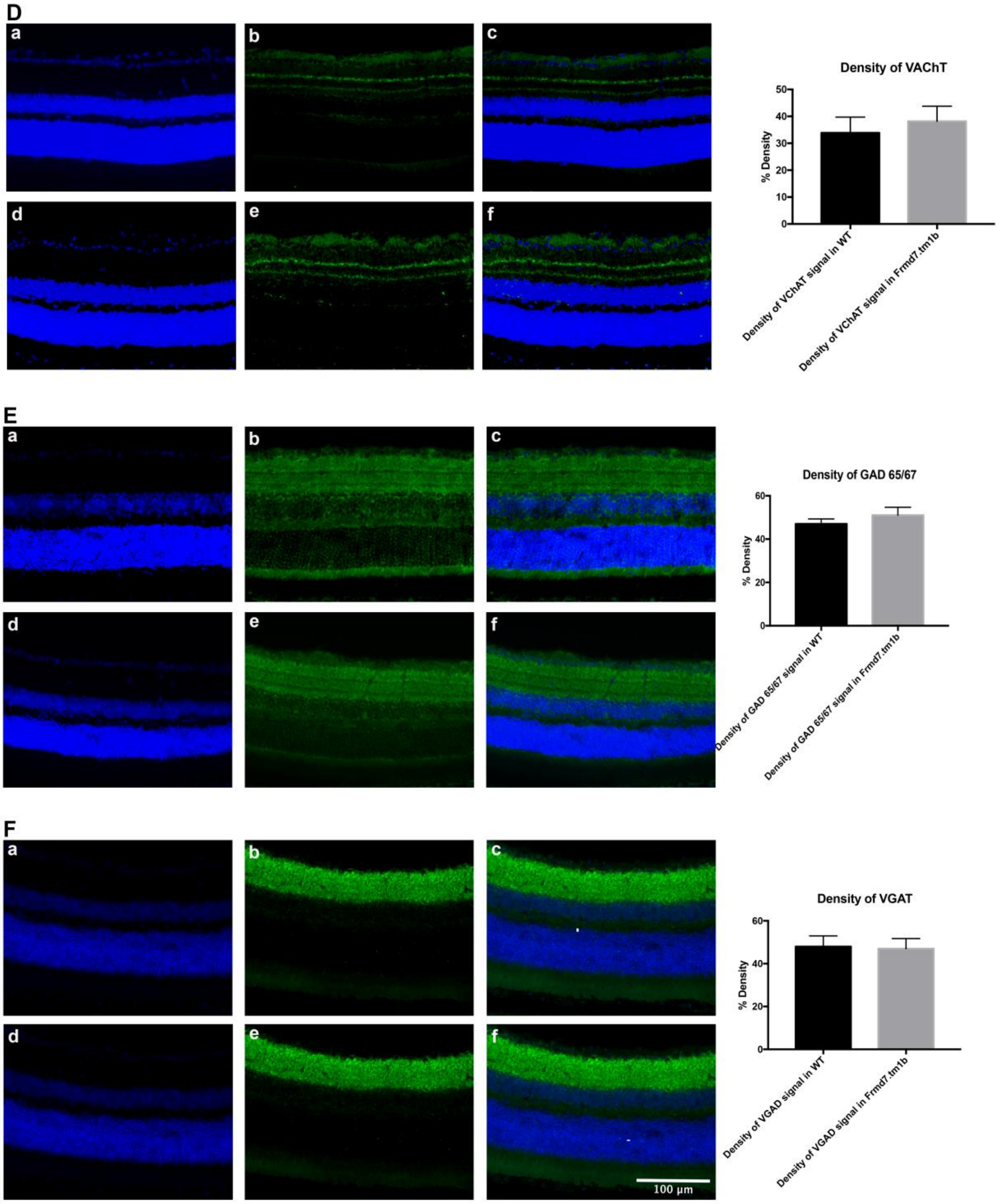

Figure S3 Density of synaptic markers in the mouse retina. Frozen retinal sections from adult male wild type littermate (n=5) (a-c) and Frmd7$^{.tm1b}$ transgenic mice (n=6) (d-f) were subjected to immunofluorescence staining against different synaptic markers. Choline Acetyltransferase (ChAT, red) (A), Synaptophysin (Abcam; Ab8049, 1:500, FITC) (B), Post-Synaptic Density (PSD96) (FITC) (C), Vesicular Acetylcholine Transporter (VChAT) (FITC) (D), Glutamic Acid Decarboxylate (GAD 65/67) (FITC) (E), and Vesicular GABA Transferase (VGAT) (FITC) (F). Sections stained with a secondary antibody only (anti-goat IgG 568 made in Donkey) used as controls. (4', 6-diamidino-2-phenylindole) (DAPI) (blue) was used as a nuclear counterstain. Percentage relative quantification of each marker is also shown. Scale bar = 100 µm, error bars = +SEM. Significance of p values calculated using a non-parametric t-test.

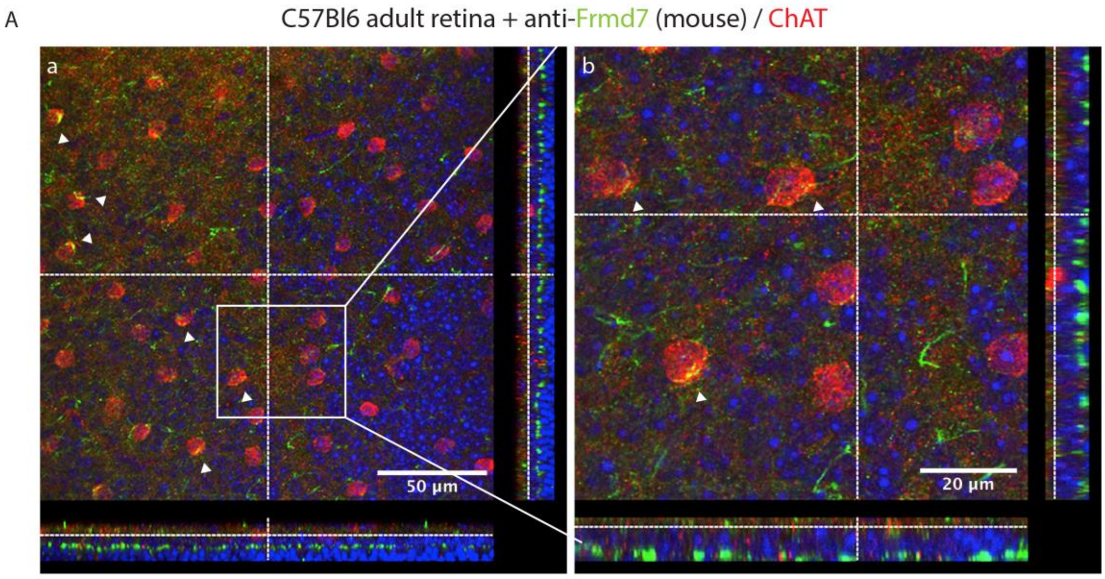

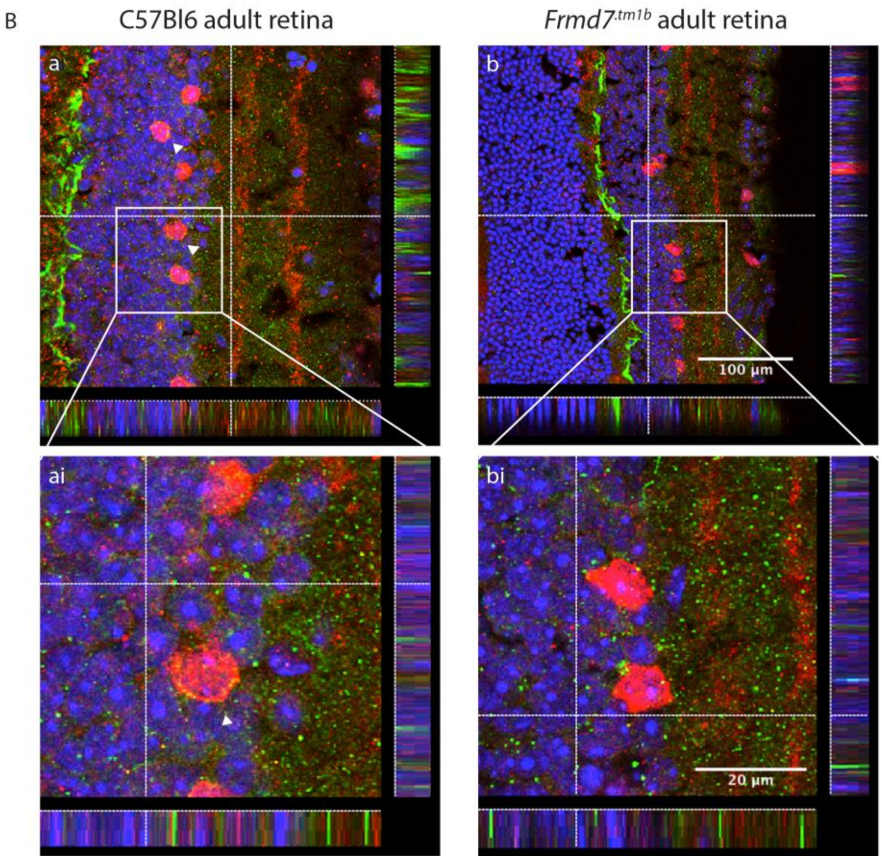

Figure S4. Wholemount retina immunofluorescence staining of Frmd7-expressing starburst amacrine cells. Confocal images of adult C57/BL6 wild type wholemount retina (n=3) subjected to immunofluorescence staining with the anti-Frmd7 antibody raised against the murine Frmd7 protein (1:100, FITC) and an anti-Choline acetyltransferase (ChAT) antibody (Merch Millipore; A144P, 1:500, red) shown in a top view. Side views are shown on the bottom and right-hand sides (A). Side views of confocal images of adult C57/BL6 and *Frmd7.tm1b* retinas subjected to immunofluorescence with anti-Frmd7 (FITC) and anti-ChAT antibodies (red) (B). (4', 6-diamidino-2-phenylindole) (DAPI) (blue) was

used as a nuclear counterstain. White arrows indicate Frmd7 and ChAT proteins colocalization. Scale bar = 20, 50 and 100 µm.

Table S1. Primers for qPCR.

Ex2F	*Frmd7* exon2	CGCTCTTCAACCTGAGCTG	55°C, 60 sec	35
Ex3F	*Frmd7* exon3	GGAACTTCTGAAGCCCATAA	55°C, 60 sec	35
Ex3R	*Frmd7* exon3	CTTGCTTCGTTATGGGCTTC	55°C, 60 sec	35
Ex4R	*Frmd7* exon4	AGTTCTTCCCGAAGATGTCC	55°C, 60 sec	35
Ex5R	*Frmd7* exon5	ATGTGCGACACCATTAAAGC	55°C, 60 sec	35
En2R	*LacZ* reporter casette	AACTCAGCCTTGAGCCTCTG	55°C, 60 sec	35
LacZF1	*LacZ* reporter cassette	TTCAACATCAGCCGCTACCAG	55°C, 60 sec	35
LacZR1	*LacZ* reporter cassette	CACCACGCTCATCGATAATTT	55°C, 60 sec	35
LacZR2	*LacZ* reporter casette	TTTCAGGTTCAGGGGGAGGRGRG	55°C, 60 sec	35

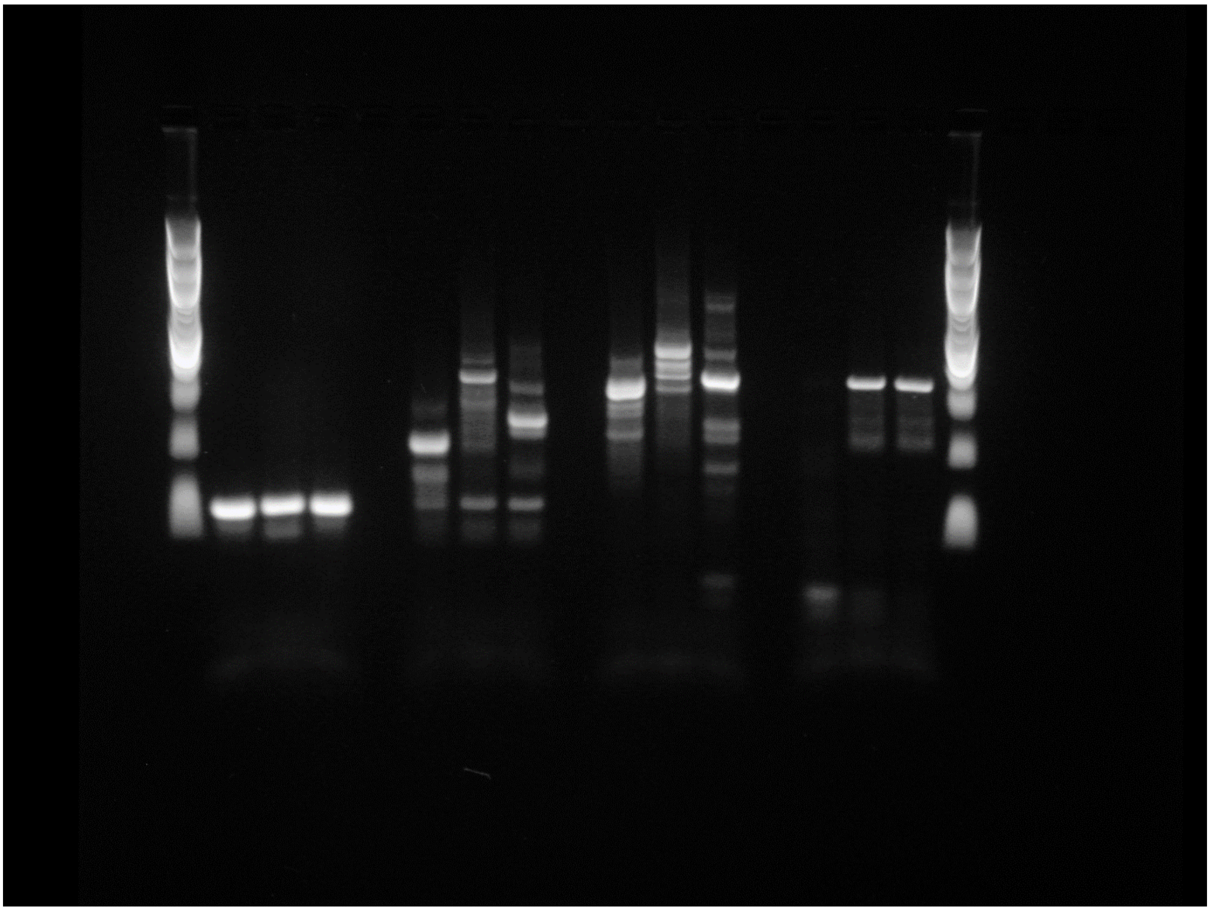

Figures S5. qPCR full gel for Figure 1 (transcript) – P1, P3, P4 and P7.

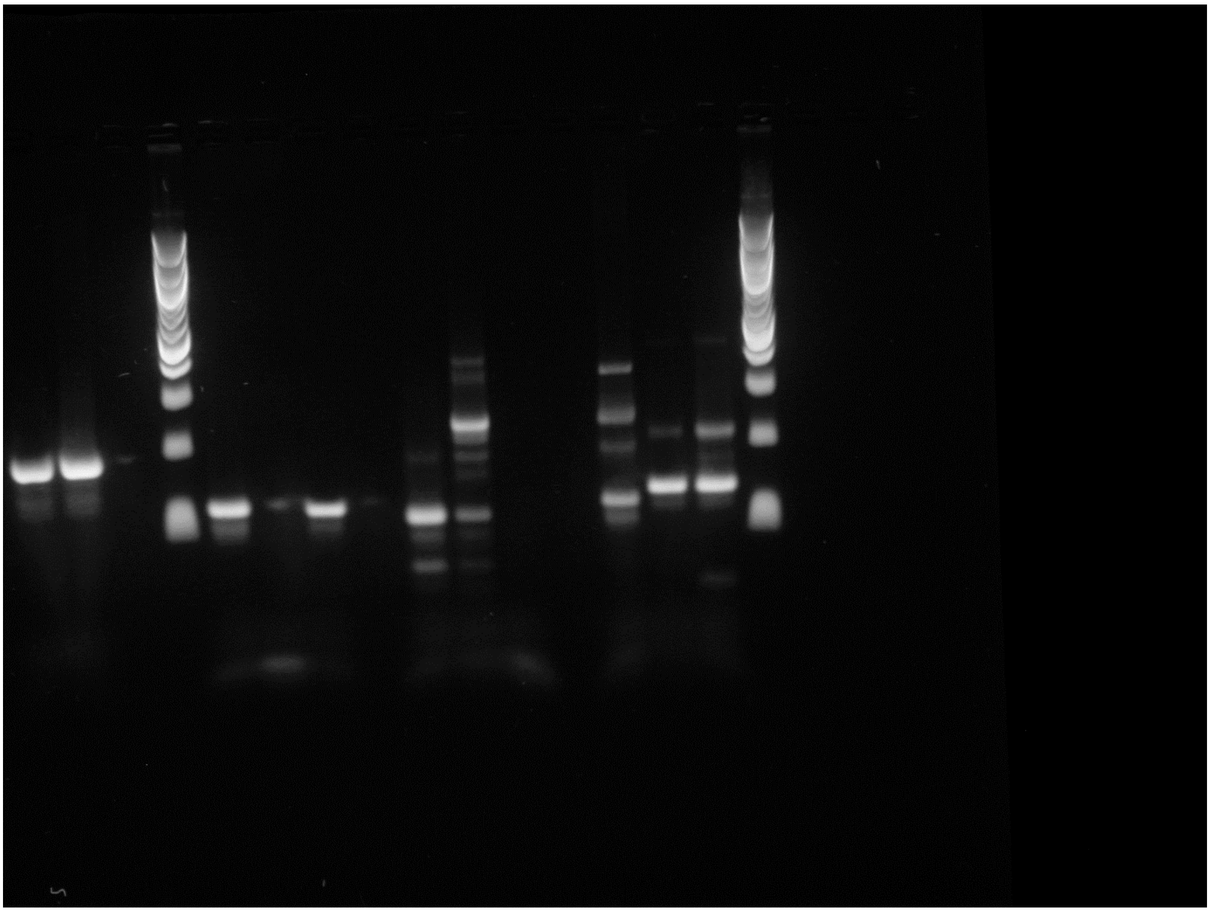

Figures S6. qPCR full gel for Figure 1 (transcript) – P2 and P5.
